# Risk factor analysis for inaccurate pre-operative MRI staging in rectal cancer

**DOI:** 10.1186/s12885-020-06761-0

**Published:** 2020-03-27

**Authors:** Zerong Cai, Xiaoyu Xie, Yufeng Chen, Zexian Chen, Wuteng Cao, Khamis Salem Saeed Saad, Yifeng Zou, Ping Lan, Xiaojian Wu

**Affiliations:** 1 Department of Colorectal Surgery, the Sixth Affiliated Hospital, Sun Yat-sen University; Guangdong Institute of Gastroenterology, Guangdong Provincial Key Laboratory of Colorectal and Pelvic Floor Diseases, the Sixth Affiliated Hospital, Sun Yat-sen University, 510655, Guangzhou, People’s Republic of China; 2 Department of Oncology, the Sixth Affiliated Hospital, Sun Yat-sen University, Guangzhou, People’s Republic of China; 3 Department of Radiology, the Sixth Affiliated Hospital, Sun Yat-sen University, Guangzhou, People’s Republic of China

**Keywords:** Rectal cancer, TNM stage, MRI

## Abstract

**Background:**

Various tumor characteristics might lead to inaccurate local MRI-defined stage of rectal cancer and the purpose of this study was to explore the clinicopathological factors that impact on the precision pre-treatment MRI-defined stage of rectal cancer.

**Methods:**

A retrospectively analysis was conducted in non-metastatic rectal cancer patients who received radical tumor resection without neoadjuvant treatment during 2007–2015 in the Sixth Affiliated Hospital of Sun Yat-sen University. Clinical T stage and N stage defined by pelvic enhanced MRI and pathological stage were compared and patients were subdivided into accurate-staging, over-staging and under-staging subgroups. Logistic regressions were used to explore risk factors for over-staging or under-staging.

**Results:**

Five hundred fifty-one cases of patients were collected. Among them, 109 cases (19.4%) of patients were over-T-staged and 50 cases (8.9%) were under-T-staged, while 78 cases (13.9%) were over-N-staged and 75 cases (13.3%) were under-N-staged. Logistic regression suggested that pre-operative bowel obstruction was risk factor for over-T-staging (OR = 3.120, 95%CI: 1.662–5.857, *P* < 0.001) as well as over-N-staging (OR = 3.494, 95%CI: 1.797–6.794, *P* < 0.001), while mucinous adenocarcinoma was a risk factor for under-N-staging (OR = 4.049, 95%CI: 1.876–8.772, *P* < 0.001). Patients with larger tumor size were at lower risk for over-T-staging (OR = 0.837, 95%CI: 0.717–0.976, *P* = 0.024) and higher risk for over-N-staging (OR = 1.434, 95%CI: 1.223–1.680, *P* < 0.001).

**Conclusion:**

Bowel obstruction, mucinous adenocarcinoma and tumor size might have impact on the pre-operative MRI T staging or N staging of rectal cancer. Our results reminded clinicians to assess clinical stage individually in such rectal cancer patients.

## Background

Rectal cancer is one of the most common gastrointestinal malignancies worldwide and it accounts for more than 50% of colorectal cancer in Asia. In the past decades, incidence of rectal cancer in China increased [1]. Treatment methods including pre-operative chemotherapy plus or not plus radiotherapy and total mesorectal excision plus radical tumor resection depends on the initial clinical stage in rectal cancer especially in those with tumor that extend beyond the rectal wall or lymph node metastatic disease [2]. Moreover, new strategy of transanal minimally invasive and local resection surgery was reported to be applicable in some selected patients with small superficial tumor according their pre-treatment TNM stage [3]. Therefore, precise local staging is crucial for appropriate initial treatment, which will significantly reduce local recurrence and improve prognosis.

Magnetic resonance imaging (MRI) has a high resolution of soft tissue and spatial resolution. These properties of MRI make it suitable to assess involvement of rectal tumor into the circumferential resection margin, relationship of the tumor to the anal sphincter and any suspicious metastatic lymph node [4]. It was reported MRI to be 87% (95%CI: 81–92%) sensitive and 75% (95%CI: 68–80%) specific for T stage and 77% (57–90%) sensitive and 71% (59–81%) specific for N stage, according to a meta-analysis included 21 articles regarding the accuracy of pelvic MRI in rectal cancer [5]. In our previous studies, we have shown that the tumor shrink evaluated by MRI could predicted pathological regression in locally advanced rectal cancer patients receiving neoadjuvant treatment [6, 7]. However, there was few article investigating factors that associated with over-staging or under-staging in the assessment of local stage by MRI, while tumor parameters such as tumor size, bowel obstruction, pathological subtype may influence the tumor morphology and tumor infiltration into the mesorectal lymph node.

Therefore, we retrospectively collected data of rectal cancer patients who received pelvic MRI scan and underwent total mesorectal excision plus radical tumor resection without neoadjuvant treatment, to explore the clinicopathological impact factors that would disturb precise clinical T staging or N staging in rectal cancer patients, which would lead to more precise treatment in rectal cancer.

## Methods

### Patients population

This study was approved by the Institutional Review Board of the Sixth Affiliated Hospital of Sun Yat-sen University. Data of patients who were diagnosed with rectal cancer in the Sixth Affiliated Hospital of Sun Yat-Sen University from October, 2007 to January, 2015 were retrospectively collected according to the following criteria. Inclusion criteria: (1) Patients were pathological diagnosed with primary adenocarcinoma including classic adenocarcinoma and mucinous adenocarcinoma. (2) Patients received pelvic contrast-enhanced MRI in 2 weeks before surgery. (3) Patients were with resectable lesion without distant metastatic disease according to the chest-abdominal-pelvic contrast enhanced computed tomography scan or laparotomy. (4) Patient underwent low anterior rectal resection or abdominoperineal excision and total mesorectal excision by open or laparoscopic operation and pathological diagnosed TNM I/II/III stage disease. Exclusion criteria: (1) Patients received anti-tumor treatment included chemotherapy, radiotherapy or targeted therapy prior to surgery. (2) Patients were accompanied with any other malignancy. (3) Patients had a history of pelvic infectious disease or were pregnant. (4) Patients loss their pathological or MRI information. Reasons for patients did not received neoadjuvant treatment included: (1) Patients who were diagnosed T_0-2_ N_0_ clinical stage by MRI; (2) Patients refused to receive any chemotherapy or radiotherapy because of financial difficulty or any other personal reason; (3) Patients have chemotherapy contraindication such as too old age, history of 3/4 grade chemotherapy toxicity or allergy to chemotherapy regent; (4) Patients have definite or relative emergency surgical indications such as bowel obstruction, uncontrolled digestive tract bleeding.

### Patients’ characteristics and MRI assessment

Patients’ clinicopathological characteristics including gender, age, BMI, history of diabetes mellitus, pre-treatment serum CEA and CA199 level, present of bowel obstruction, pre-treatment white blood cell (WBC), hemoglobin (HB) and plate (PLT) level, tumor size of lesion according to the MRIs, location of tumor and pathological information were collected from the database of the Sixth Affiliated Hospital of Sun Yat-sen University. Bowel obstruction is diagnosed on the basis of any of the following criteria: plain X-ray or contrast studies indicate obstruction, patient presented with abdominal pain, vomiting, abdominal distension and absence of gas and stool for more than 24 h. All resected specimens were examined by two experienced pathologists and the information of histological subtype, tumor differentiation, mismatch repair (MMR) status and RAS mutations were also collected. Patients’ prognosis information including overall survival and disease-free survival was obtained from the Colorectal Cancer Follow-up Database in the Sixth Affiliated Hospital of Sun Yat-sen University.

Pelvic MRIs including T2WI, T1WI, DWI and contrast agent-enhanced LAVA Flex were performed using a 1.5 T imaging unit (Optima MR360 GE Medical Systems) according to the protocols previously described [8]. All the MR images were reviewed by two gastrointestinal radiologists separately who were both only aware that the MRIs were from primary rectal cancer patients and blinded to any other clinicopathological information. The MRI-defined T staging and N staging category criteria were descripted as previously described [8].

### Definition of subgroups

Patients were divided into groups including accurate-staging (clinical stage = pathological stage), over-staging (clinical stage > pathological stage) and under-staging (clinical stage < pathological stage), according to the comparison between MRI and pathological T/N stage.

### Statistical analysis

Mean ± Standard Deviations was used to present continuous variables and number (percentage) for categorical data. One-Way ANOVA and Chi-square test were performed when compared between different subgroups and the Least Significant Difference was employed to further test for significant difference. Univariate and multivariate logistic regression models were utilized to explore risk factors for over-staging or under-staging. Kaplan-Meier method was used to estimate patients’ survival between subgroups. All statistical analyses were performed by SPSS software (version 19.0, Chicago, IL) and *P* value less than 0.05 was considered statistically significant.

## Results

### Patients’ characteristics

A total of 551 rectal cancer patients who received low anterior rectal resection (*n* = 488, 88.6%) or abdominoperineal excision (*n* = 63, 11.4%) without pre-operative treatment were enrolled in this study. As showed in Table 1, the average age of the patients was 59.7 ± 13.4 and 233 cases (42.3%) of them were female. Of all patients, 33 cases (6.1%) of patients had comorbidity of diabetes mellitus and 67 cases of patients (12.5%) presented with bowel obstruction when diagnosed with rectal cancer. According to the pathological assessment of the resected specimen, 44 cases (8.0%) of mucinous adenocarcinoma and 39 cases (7.6%) of grade 3/4 differentiation tumor were found. Sixty-one cases (12.2%) of T_1_ stage tumor, 123 cases (22.3%) of T_2_ stage tumor, 334 cases (60.6%) of T_3_ stage tumor and 27 cases (4.9%) of T_4_ stage tumor were diagnosed and 178 cases (32.3%) of patients were diagnose N_+_ (Data not shown in Table 1). Comparing the MRI-defined clinical stage and pathological T/N stage, 109 cases (19.4%) of over-T-stage, 50 cases (8.9%) of under-T-stage, 78 cases (13.9%) of over-N-stage and 75 cases (13.3%) of under-T-stage were found.

**Table 1:** Characteristics of patients in subgroups of accurate-T-staging, over-T-staging and under-T-staging

Characteristics	Total	Accurate-T-staging	Over-T-staging	Under-T-staging	*P* Value
Cases	551	392 (69.6%)	109 (19.4%)	50 (8.9%)	
Gender					0.633
Female	233 (42.3%)	169 (43.1%)	46 (42.2%)	18 (36%)	
Male	318 (57.7%)	223 (56.9%)	63 (57.8%)	32 (64%)	
Age	59.7 ± 13.4	60.2 ± 12.9	57.3 ± 14.4	61.3 ± 14.4	0.102
BMI	22.5 ± 4.19	22.5 ± 4.4	22.5 ± 3.8	22.5 ± 3.4	0.998
Diabetes Mellitus					0.157
No	507 (93.9%)	360 (93%)	99 (94.3%)	48 (100%)	
Yes	33 (6.1%)	27 (7%)	6 (5.7%)	0 (0%)	
CEA	2.73 (0–255)	2.7 (0–255)	2.7 (0–197)	4.5 (1–81)	0.62
CA199	10.2 (0–2188)	10.64 (0–2188)	7.59 (0–1156)	10.35 (0–117)	0.001
Bowel Obstruction					0.008
No	471 (87.5%)	350 (89.7%)	88 (79.3%)	44 (89.8%)	
Yes	67 (12.5%)	40 (10.3%)	23 (20.7%)	5 (10.2%)	
WBC	6.53 ± 1.93	6.54 ± 1.91	6.53 ± 2.11	6.48 ± 1.71	0.984
HB	124.3 ± 21.5	125.0 ± 19.5	123.4 ± 24.4	121.1 ± 28.7	0.433
PLT	234.5 ± 78.4	237.6 ± 80.1	227.2 ± 74.5	226.4 ± 72.7	0.355
ALB	41.5 ± 6.30	41.6 ± 6.10	40.8 ± 6.96	42.2 ± 6.37	0.381
Tumor Location					0.186
Upper	217 (39.4%)	163 (41.6%)	37 (33.9%)	17 (34%)	
Middle	141 (25.6%)	101 (25.8%)	24 (22%)	16 (32%)	
Low	193 (35%)	128 (32.7%)	48 (44%)	17 (34%)	
Tumor Size (cm)	4.07 ± 1.69	4.22 ± 1.70	3.76 ± 1.70	3.60 ± 1.37	0.005^*^
Histological Subtype					1
Classic Adenocarcinoma	505 (92%)	359 (92.1%)	100 (91.7%)	46 (92%)	
Mucinous Adenocarcinoma	44 (8%)	31 (7.9%)	9 (8.3%)	4 (8%)	
Differentiation					0.824
Grade 1/2	471 (92.4%)	337 (92.1%)	91 (94.8%)	43 (89.6%)	
Grade 3/4	39 (7.6%)	29 (7.9%)	5 (5.2%)	5 (10.4%)	
MMR Status					0.823
pMMR	350 (94.1%)	17 (6.6%)	4 (4.8%)	1 (3.1%)	
dMMR	22 (5.9%)	239 (93.4%)	80 (95.2%)	31 (96.9%)	
Ras Mutation					0.322
No	496 (90%)	355 (90.6%)	99 (90.8%)	42 (84%)	
Yes	55 (10%)	37 (9.4%)	10 (9.2%)	8 (16%)	

*Abbreviations*: *BMI* body mass index, *CEA* carcinoembryonic antigen, *WBC* white blood cell, *HB* hemoglobin, *PLT* plate, *ALB* albumin, *pMMR* mismatch repair-proficient, *dMMR* mismatch repair-deficient

^*^Post Hoc Test: Accurate-T-staging vs over-T-staging, *P* = 0.012; Accurate-T-staging vs under-T-staging, *P* = 0.015

Bowel obstruction was associated with over-T-staging and over-N-staging, and tumor size was associated with over-T-staging, under-T-staging and over-N-staging, while mucinous adenocarcinoma was associated with under-N-staging.

We compared the clinicopathological characteristics between patients who were accurate-staging, over-staging and under-staging in terms of T stage and N stage respectively. It was suggested that patients of over-T-staging were more likely to have greater proportion of bowel obstruction (20.7% vs 10.3%, *P* < 0.05) and smaller tumor size (3.76 vs 4.22, *P* < 0.05) when compared with those of accurate-T-staging as showed in Table 1. And patients who were under-T-staging were characterized with smaller tumor size (3.60 vs 4.22, *P* < 0.05). It was suggested in Table 2 that patients who were over-N-staging were more likely to characterized with more proportion of bowel obstruction (31.2% vs 8.5%, *P* < 0.05), higher PLT level (257.1 vs 226.9, *P* < 0.05) and larger tumor size (5.59 vs 4.11, *P* < 0.05) when compared with those of accurate-N-staging. Patients with under-N-staging were more likely to have higher PLT level (253.5 vs 226.9, *P* < 0.05) and have a greater proportion of mucinous adenocarcinoma (20.3% vs 5.8%, *P* < 0.001).

**Table 2:** Characteristics of patients in subgroups of accurate-N-staging, over-N-staging and under-N-staging

Characteristics	Accurate-N-staging	Over-N-staging	Under-N-staging	*P* Value
Cases	398 (70.7%)	78 (13.9%)	75 (13.3%)	
Gender				
Female	171 (43%)	31 (39.7%)	31 (41.3%)	0.853
Male	227 (57%)	47 (60.3%)	44 (58.7%)	
Age	60.0 ± 13.2	58.2 ± 14.3	58.7 ± 14.4	0.514
BMI	22.5 ± 3.52	21.8 ± 4.31	23.3 ± 6.44	0.048
DM				0.709
No	366 (93.6%)	73 (96.1%)	68 (93.2%)	
Yes	25 (6.4%)	3 (3.9%)	5 (6.8%)	
CEA	2.69 (0–117)	3.08 (1–255)	2.94 (0–81)	0.152
CA199	10.27 (0–2188)	9.57 (0–322)	10.59 (0–723)	0.491
Bowel Obstruction				< 0.001
No	354 (91.5%)	53 (68.8%)	64 (86.5%)	
Yes	33 (8.5%)	24 (31.2%)	10 (13.5%)	
WBC	6.37 ± 1.82	7.31 ± 2.44	6.56 ± 1.71	0.924
HB	125.8 ± 20.3	119.5 ± 21.7	121.3 ± 26.3	0.053
PLT	226.9 ± 75.3	257.1 ± 91.7	253.5 ± 72.8	0.001^*^
ALB	41.6 ± 6.74	40.5 ± 5.12	42.2 ± 4.58	0.216
Tumor Location				0.6
Upper	152 (38.2%)	35 (44.9%)	30 (40%)	
Middle	100 (25.1%)	22 (28.2%)	19 (25.3%)	
Low	146 (36.7%)	21 (26.9%)	26 (34.7%)	
Tumor Size (cm)	4.11 ± 3.48	5.59 ± 3.11	4.11 ± 1.71	< 0.001^**^
Histological Subtype				< 0.001
Classic adenocarcinoma	374 (94.2%)	72 (92.3%)	59 (79.7%)	
Mucinous adenocarcinoma	23 (5.8%)	6 (7.7%)	15 (20.3%)	
Differentiation				0.298
Well/Moderate	349 (93.3%)	68 (90.7%)	54 (88.5%)	
Poor	25 (6.7%)	7 (9.3%)	7 (11.5%)	
MMR Status				0.3
pMMR	251 (94.7%)	52 (89.7%)	47 (95.9%)	
dMMR	14 (5.3%)	6 (10.3%)	2 (4.1%)	
Ras Mutation				0.483
No	355 (89.2%)	73 (93.6%)	68 (90.7%)	
Yes	43 (10.8%)	5 (6.4%)	7 (9.3%)	

*Abbreviations*: *BMI* body mass index, *CEA* carcinoembryonic antigen, *WBC* white blood cell, *HB* hemoglobin, *PLT* plate, *ALB* albumin, *pMMR* mismatch repair-proficient, *dMMR* mismatch repair-deficient

^*^Post Hoc Test: Accurate-N-staging vs over-N-staging, *P* = 0.002; Accurate-N-staging vs under-N-staging, *P* = 0.011

^**^Post Hoc Test: Accurate-N-staging vs over-N-staging, *P* < 0.001

Bowel obstruction increased patients’ risk for over-T-staging and over-N-staging, and larger tumor size was a protective factor for over-T-staging and a risk factor for over-N-staging, while mucinous adenocarcinoma increased risk for under-N-staging in rectal cancer.

We used logistic regression to explore pre-operative clinicopathological characteristics that impact on over-staging or under-staging in rectal cancer in terms of T stage and N stage respectively. Multivariate logistic regression model was adjusted for confounding factors including CEA, CA199, BMI, WBC, HB, PLT, ALB, Tumor Location (Upper, Middle, Low), Histological Subtype (Classic adenocarcinoma, Mucinous adenocarcinoma), Tumor Size and Bowel Obstruction (No, Yes).As showed in Tables 3 and 4, bowel obstruction were risk factors for over-T-staging in rectal cancer (OR = 3.120, 95%CI: 1.662–5.857, *P* < 0.001), while larger tumor size were protective factor for over-T-staging (OR = 0.837, 95%CI: 0.717–0.976, *P* = 0.024). Patients who with higher HB level were at lower risk for under-T-staging (OR = 0.981, 95%CI: 0.964–0.998, *P* = 0.027) and those with higher ALB level were at higher risk for under-T-staging (OR = 1.068, 95%CI: 1.007–1.133, *P* = 0.029). Risk factors for over-N-staging included higher WBC level (OR = 1.174, 95%CI: 1.007–1.368, *P* = 0.041), larger tumor size (OR = 1.434, 95%CI: 1.223–1.680, *P* < 0.001) and bowel obstruction (OR = 3.494, 95%CI: 1.797–6.794, *P* < 0.001), while mucinous adenocarcinoma (OR = 4.049, 95%CI: 1.876–8.772, *P* < 0.001) was significant risk factor for under-N-staging.

**Table 3:** Multivariate logistic regression model of risk factors for over-T-staging and under-T-staging in rectal cancer

Characteristics	Over-T-staging			Under-T-staging		
	OR	95% CI	*P* Value	OR	95% CI	*P* Value
CEA	1.001	0.988–1.014	0.907	1.008	0.995–1.02	0.214
CA199	0.999	0.996–1.002	0.374	0.998	0.993–1.004	0.557
BMI	1.003	0.947–1.063	0.906	0.997	0.916–1.085	0.944
WBC	1.011	0.884–1.156	0.874	1.055	0.873–1.275	0.578
HB	0.999	0.986–1.012	0.862	0.981	0.964–0.998	0.027
PLT	0.998	0.994–1.001	0.163	0.998	0.994–1.003	0.522
ALB	0.98	0.942–1.019	0.307	1.068	1.007–1.133	0.029
Tumor Location	0.644	0.382–1.087	0.186			0.923
Upper	0.656	0.368–1.168	0.1	1.05	0.49–2.251	0.901
Middle			0.152	1.175	0.524–2.635	0.696
Low	1			1		
Histological Subtype			0.53			
Classic adenocarcinoma	1			1		0.638
Mucinous adenocarcinoma	1.316	0.558–3.106		1.319	0.416–4.184	
Tumor Size	0.837	0.717–0.976	0.024	0.808	0.646–1.012	0.064
Bowel Obstruction			< 0.001			
No	1			1		
Yes	3.12	1.662–5.857		1.267	0.455–3.528	0.65

*Abbreviations*: *CEA* carcinoembryonic antigen, *BMI* body mass index, *WBC* white blood cell, *HB* hemoglobin, *PLT* plate, *ALB* albumin

**Table 4:** Multivariate logistic regression model of risk factors for over-N-staging and under-N-staging in rectal cancer

Characteristics	Over-N-staging			Under-N-staging		
	OR	95% CI	*P* Value	OR	95% CI	*P* Value
CEA	1.012	1–1.024	0.06	1.002	0.988–1.015	0.792
CA199	0.996	0.99–1.001	0.147	1	0.998–1.002	0.847
BMI	0.953	0.88–1.031	0.232	1.063	1–1.129	0.05
WBC	1.174	1.007–1.368	0.041	1.038	0.888–1.214	0.641
HB	0.993	0.977–1.009	0.383	0.993	0.978–1.009	0.411
PLT	1	0.996–1.003	0.838	1.002	0.998–1.006	0.33
ALB	1.002	0.953–1.053	0.953	1.027	0.976–1.082	0.306
Tumor Location			0.738			0.875
Upper	1.216	0.629–2.351	0.56	1.157	0.613–2.184	0.653
Middle	1.322	0.638–2.739	0.453	1.002	0.493–2.036	0.995
Low	1			1		
Histological Subtype			0.285			< 0.001
Classic adenocarcinoma	1			1		
Mucinous adenocarcinoma	0.533	0.168–1.686		4.049	1.876–8.772	
Tumor Size	1.434	1.223–1.680	< 0.001	0.953	0.806–1.126	0.572
Bowel Obstruction			< 0.001			0.596
No	1			1		
Yes	3.494	1.797–6.794		1.243	0.556–2.776	

*Abbreviations*: *CEA* carcinoembryonic antigen, *BMI* body mass index, *WBC* white blood cell, *HB* hemoglobin, *PLT* plate, *ALB* albumin

Neither over-staging nor under-staging affected the prognosis of rectal cancer patients.

**Fig. 1:**
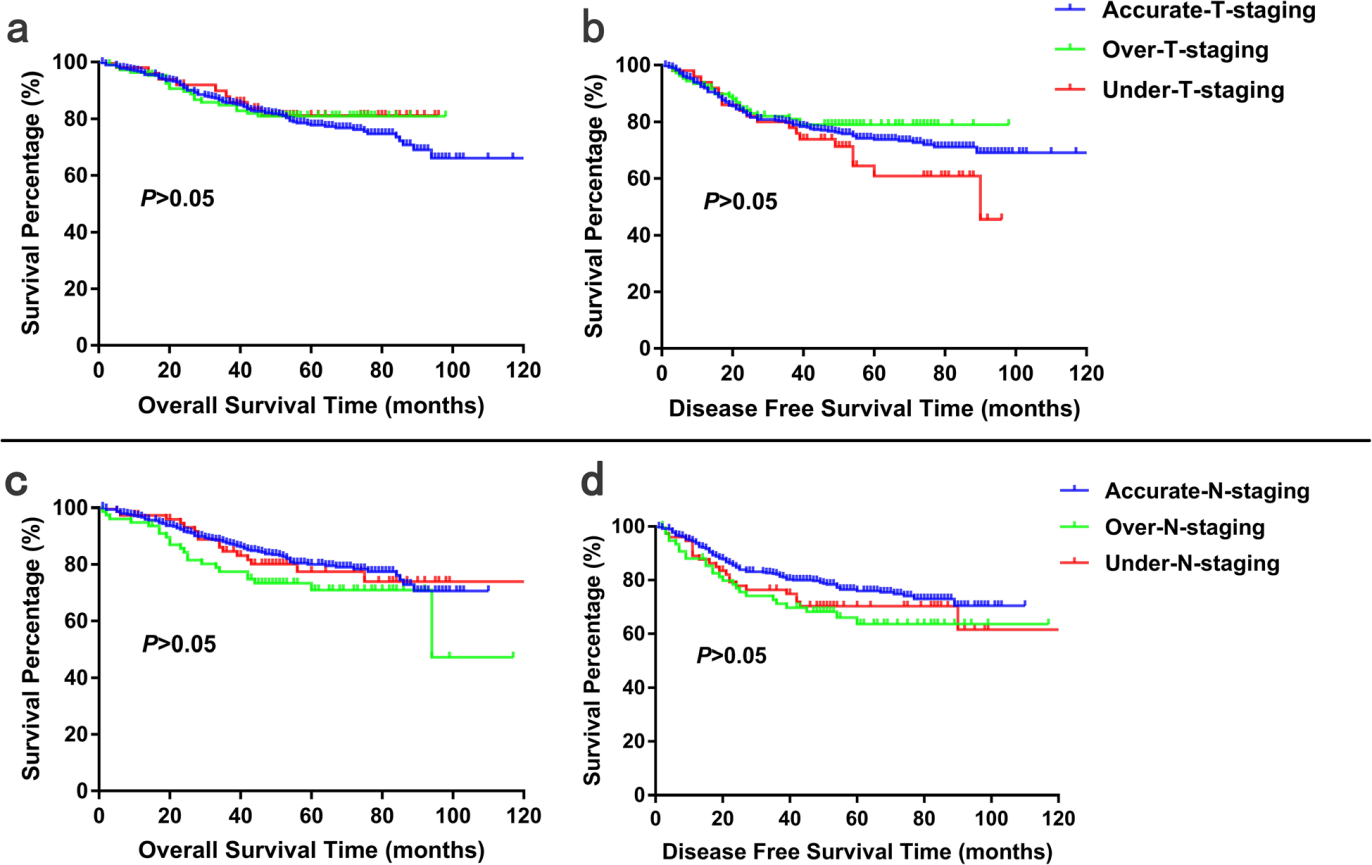
Kaplan-Meier survival curve of rectal cancer patients by subgroups of accurate staging, over-staging and under-staging in terms of T stage and N stage. Log-rank test showed no significant difference of overall survival (**a**, **c**) and disease free survival (**b**, **d**) for patients of accurate staging, over-staging and under-staging

We compared the overall survival and disease free survival between accurate-staging, over-staging and under-staging subgroups by Kaplan-Meier analysis. As showed in Fig. 1, it was suggested that no statistically significant difference of overall survival or disease free survival was found between these subgroups (*P* > 0.05).

## Discussion

In this study, we conduct a retrospectively analysis to investigate the impact of the clinicopathological factors on the over-staging or under-staging in rectal cancer patients, which were grouped based on the comparison between MRI-defined clinical stage and pathological stage. It was revealed that patients who presented with bowel obstruction were at higher risk of being over-T-staged and over-N-staged. In addition, patients with larger tumor size were at lower risk of being over-T-staged but a higher risk of being over-N-staged, and patients with mucinous adenocarcinoma were more likely to be under-N-staged.

In the recent decade, new innovations and advances have been made in the treatments of rectal cancer especially locally advanced cancer to avoid recurrence and improve patients’ survival [3]. Among these advances, chemoradiotherapy leads to significant tumor regression and results in more sphincter preservation in rectal cancer [2]. A transanal minimally invasive treatment by local resection surgery could be applicable for patients diagnosed with early T stage and negative N stage tumor. Precise pre-treatment clinical TNM staging could avoid unnecessary excessive treatment in patients who were over-staged and insufficient treatment in those who were under-staged. Recommended by NCCN clinical practice guideline of rectal cancer [2, 9], pelvic MRI is essential assessment for rectal cancer before treatment strategy been made since it can diagnose rectal wall laminar structure and show detail of the relationship of the tumor with the mesorectal fascia and surrounding organs [10]. However, whether the edema and chronic fibrosis of the bowel wall, which were caused by bowel obstruction, will interfere the accuracy of MRI in distinguishing the laminar structure remain unknown. In this study, we found that patients who presented with bowel obstruction were at higher risk to be over-stage when assessing the T stage by MRI, and N stage was also likely to be over-staged. The edema of the bowel wall and enlargement of the mesorectal lymph node caused by bowel obstruction would confuse the tumor infiltration with intestinal inflammation, which might result in inappropriate treatment strategy. This result will highlight the necessity to reevaluation the T stage and N stage in obstructing cases.

In addition, our result demonstrated that patients with smaller tumor were at higher risk of being over-T-staged and those with larger tumor were at higher risk of being over-N-staged. It was suggested that patients with smaller rectal tumor were associated with favor survival [11] and they were often have earlier T stage. When assessing the T stage of those patients with small and early T stage tumor, MRI were more likely to over-stage in patients with T_1_ or T_2_ disease, as the band of fibrous tissue extend beyond the muscularis propria may be difficult to distinguish from tumor [12]. On the other hand, patients with large tumor were more likely to have bowel obstruction and thus the enlargement lymph node caused by inflammatory would lead to over-N-staging in patients with larger rectal tumor.

Mucinous adenocarcinoma is a common histological variant of rectal cancer which comprises about 18% of rectal cancers [13]. It is characterized by abundant extracellular mucin that constitutes more than 50% of the tumor mass and demonstrated with a different molecular pattern when compared with adenocarcinoma [14, 15]. Rectal mucinous adenocarcinoma manifested a distinct disease behavior such as poor response to neoadjuvant chemoradiotherapy and unfavorable prognosis, according to a meta-analysis conclusion [16]. It can often be identified by MRI from the large, signal-intense mucin pools with high sensitivity and specificity [17]. However, the influence of mucinous subtype on the accuracy in N stage of rectal cancer by MRI has not been reported. In our study, it was demonstrated that mucinous adenocarcinoma were risk factors for under-N-staging. The lymphatics can take up fluid mucus with tumor cells into the peritoneal cavity and help with infiltrate into the regional lymph nodes [18]. This aggressive disease behavior will made it difficult to diagnose N_+_ disease when we evaluate metastatic lymph node by their sizes, which lead to under-N-stage in rectal mucinous adenocarcinomas. Therefore, clinicians should stay vigilant of lymph node status when diagnosing rectal mucinous carcinoma and distinct evaluation criterion should be established for lymph node metastasis assessment in mucinous rectal adenocarcinoma.

We tried to evaluation the impact of over or under staging on patients’ prognosis. However, no significant impact of over-stage or under-stage on patients’ prognosis was found according to the Kaplan-Meier analysis. Only 119 cases of patients (69.2%) with TNM stage III disease completed adjuvant chemotherapy according to our data (not showed in the result). Besides the compliance to receive adjuvant treatment, characteristics such as pathological stage, tumor size and histological type were varied between patients of under-staged, accurate-staged and over-staged. Therefore, the real influence of inaccurate stage of patients was difficult to examine and would be analyzed in our further work.

There were limitations in this study. First, this is a retrospective single-center study and we included rectal patients who did not receive pre-operative chemotherapy to avoid the pathological down-stage by chemotherapy. Therefore, an inevitable selection bias still exist due to the study design. Neoadjuvant chemoradiotherapy was the standard treatment for local advanced rectal cancer (T_3+_N_0_ or T_any_N_+_) according to most guidelines. However, in order to evaluate the truly pathological stage, we had to exclude those cases who received pre-operative treatment. And the included patients might be characterized with more early stage (27.9% of patients were T_1–2_N_0_), more bowel obstruction (12.5%) and less treatment compliance to receive chemotherapy. Further investigation by a prospective multi-center cohort is still needed to prove our conclusion. Second, we excluded metastatic rectal cancers in this study because the palliative therapy in most metastatic disease would not be influenced by local T or N stage. The accuracy of MRI staging in metastatic rectal cancers still remains unknown. Likewise, patients who were MRI diagnosed T_1–2_ rectal cancer but were pathological diagnosed T_0_ disease (rectal adenoma) were also excluded due to the study design. The MRI over-staging in rectal adenoma also remain further evaluation. Third, we did not investigate the impact of under-staging and over-staging on the treatment options in rectal cancer patients in this study. The real impact of staging inaccuracy on the patients still need to be further investigated.

## Conclusion

Rectal cancer patients with bowel obstruction were at higher risk of being over-T-staged and over-N-staged. Patients with larger tumor size were at lower risk of being over-T-staged but a higher risk of being over-N-staged, and patients with mucinous adenocarcinoma were more likely to be under-N-staged. Our results would remind clinicians to be aware of precise pre-clinical stage in such cases.

## Data Availability

The datasets generated and/or analyzed during the current study are not publicly available due to data privacy according to the license for the current study, but are available from the corresponding author on reasonable request.

## Abbreviations

ALB: Albumin
BMI: Body mass index
CEA: Carcinoembryonic antigen
dMMR: Mismatch repair-deficient
HB: Hemoglobin
LSD: Least significant difference
MRI: Magnetic resonance imaging
MMR: Mismatch repair
PLT: Plate
pMMR: Mismatch repair-proficient
WBC: White blood cell
